# Inflammatory tissue reactions around aseptically loose cemented hip prostheses: A retrieval study of the Spectron EF stem with Reflection All‐Poly acetabular cup

**DOI:** 10.1002/jbm.b.35023

**Published:** 2022-01-31

**Authors:** Susann Wolf, Anne Christine Johannessen, Peter Ellison, Ove Furnes, Geir Hallan, Katharina Rogg, Kathrine Skarstein, Paul Johan Høl

**Affiliations:** ^1^ Biomatlab, Department of Orthopedic Surgery Haukeland University Hospital Bergen Norway; ^2^ National Institute of Occupational Health Oslo Norway; ^3^ Department of Clinical Medicine University of Bergen Bergen Norway; ^4^ Department of Pathology Haukeland University Hospital Bergen Norway; ^5^ The Norwegian Arthroplasty Register, Department of Orthopedic Surgery Haukeland University Hospital Bergen Norway

**Keywords:** adverse local tissue reactions, blood metal ions, cemented total hip arthroplasty, inflammation, osteolysis, wear debris

## Abstract

The cemented Spectron EF stem in combination with the cemented non‐crosslinked Reflection All‐Poly cup showed a high rate of mid‐term aseptic loosening. However, the failure mechanisms are not fully known. We assessed the inflammatory tissue reactions and wear particles in periprosthetic tissues, implant wear and blood metal ion levels in 28 patients with failed implants. Histological analysis showed a macrophage pre‐dominant pattern with randomly distributed lymphocytes, with various amounts of neutrophils and giant cells. The number of different cell types in the tissue samples from patients in the cup group and in the stem group was similar. Wear particles, mainly ZrO_2_, CoCrMo, and polyethylene particles of different sizes and shapes, were associated with macrophages/giant cells, and total particle load/mm^2^ was higher in cases of stem loosening. The Spectron EF stems were heavily worn, abraded, and polished. Stem abrasion correlated with metal ion concentrations in blood. The median polyethylene wear rate of the Reflection cups was 0.23 mm/year. The high proximal roughness of the Spectron EF stem resulted in excessive cement wear during loosening. The resulting inflammatory tissue responses to the degradation products both from the cup and the stem led to massive osteolysis and subsequent implant loosening.

## 1 INTRODUCTION

Total hip arthroplasty (THA) is a successful, cost‐effective surgical procedure to treat arthritic hip joints. One of the most commonly used cemented hip stems in Norway in the period 1998–2008 was the Spectron EF (Smith and Nephew, Memphis, TN). In 1989, the modular Spectron EF stem was introduced as a new version of the original monoblock Spectron stem with a roughened proximal third of the new stem (Ra was changed from 0.76 to 7.3 μm), but the survival rates turned out to be unexpectedly low compared to the old design.^1^ The original cemented Spectron stem, introduced in 1983, was a monoblock, collared component with a satin finish made of cobalt chromium alloy that achieved very good survival rates of 98.4% after 10 years, as reported by the Swedish Total Hip Register^2^ and showed superior longevity compared to the cemented Charnley stem.^3^ Early reports of the new design raised concerns regarding the stem survival, and this was partly attributed to the roughness of the stem.^4^, ^5^ Later, another study also found high levels of osteolysis and stem failure with the Spectron EF.^6^ The cemented Charnley, low‐friction arthroplasty, pioneered by Sir John Charnley,^7^ provides the basis of comparison to new designs in modern cemented hip arthroplasty as it has become the gold standard in THA.^8^ The Norwegian Arthroplasty Register (NAR) reported a high number of cases of mid‐term aseptic loosening of the cobalt‐chrome Spectron EF stem in combination with the reflection cemented non‐crosslinked All‐Poly cup (Smith and Nephew) compared to the Charnley THA. They identified a 3.8‐fold higher risk of revision for this combination.^9^ The wear rate of the Reflection All‐Poly cup was shown to be very high in a randomized controlled trial.^10^ A major cause for revision following THA is aseptic loosening due to osteolysis, that is, bone resorption due to an inflammatory tissue response around the implant.^11^, ^12^ Several theories on the cause of aseptic loosening have been proposed, such as micro‐motion, stress shielding, high fluid pressure, cellular activation by wear particles, endotoxin, or individual or genetic variations.^13^ However, most researchers believe that foreign body wear particles, generated from the articulating surfaces or from the bone/implant or bone/cement interface, are the main cause for peri‐prosthetic inflammation and consequently aseptic loosening. The objective of the present study was to assess the inflammatory tissue reactions and wear particles in tissues surrounding failed implants (Spectron EF stem/Reflection All‐Poly cup), implant wear and blood metal ion concentrations in patients with cup loosening only and patients with stem and cup loosening. Moreover, we aimed to answer the following questions: (a) is the inflammatory tissue response associated with the particle count in the tissue; (b) is the number of polyethylene particles higher in patients with cup loosening only; and (c) does the number of particles correlate with the blood metal ion concentration.

## 2 MATERIALS AND METHODS

### 2.1 Patients and implants

A total of 28 patients from our retrieval biobank with the same THA (Spectron EF stem and Reflection non‐crosslinked All‐Poly cup) who underwent revision hip arthroplasty from October 2007 to January 2019 (primary arthroplasty between 1997 and 2008) were included in this study. The retrieved implants with surrounding tissue and blood samples were collected from seven hospitals in Norway. The femoral stem was made of cobalt‐chromium‐molybdenum (CoCrMo). The stems included in our study varied between three different sizes (1, 2, and 3). The femoral head was made of CoCrMo (28 mm) in most cases, but two had Oxinium™ heads, one a zirconia head and one an alumina head. The acetabular cup was made of conventional, non‐crosslinked ultra‐high molecular weight polyethylene (UHMWPE) sterilized with ethylene oxide. Both stem and cup were cemented using polymethylmethacrylate (PMMA)‐based bone cement (Palacos G (Schering‐Plow), Refobacin Bone Cement (Biomet Merck) or Palacos R + G (Heraeus)), containing 15% radio‐opaque zirconium dioxide (ZrO_2_) particles. In one case, a CMW™ cement (DePuy Synthes), containing radio‐opaque barium sulphate (BaSO_4_), was used. Information about the gender of the patients, age at revision, implant duration and reason for revision was provided by the Norwegian Arthroplasty Register (Table 1).^9^ We divided the patients in two groups according to the reason for revision: Patients with cup loosening (cup group; *n =* 10) and patients with stem and cup loosening (stem group; *n =* 18) (Table 1).

**TABLE 1:** Patient data, implant duration, and reason for revision. Two patient groups are included in this study: patients with loose cup only and patients with loose stem with cup loosening. A descriptive summary of the age at revision and implant duration is given below the respective group

Case no.	Gender, age at revision (year)	Implant duration (months)	Reason for revision
*Loose cup only*			
1	M, 59	76	Loose cup
2	M, 81	92	Loose cup
3	M, 65	113	Loose cup
4	M, 67	72	Loose cup, osteolysis
5	F, 73	135	Loose cup, PE wear
6	F, 78	77	Loose cup
7	F, 74	97	Loose cup
8	M, 59	82	Loose cup
9	F, 79	150	Loose cup, wear
10	F, 64	207	Loose cup
Median	70^a^	94.5^b^	
Range	59–81	72–207	
*Loose stem and cup*			
11	M, 81	97	Loose stem, loose cup, osteolysis
12	F, 38	138	Loose stem, loose cup, osteolysis
13	M, 68	93	Loose stem, loose cup, osteolysis in acetabulum
14	F, 72	86	Loose stem, loose cup
15	M, 59	39	Loose stem, loose cup
16	M, 66	87	Loose stem, loose cup
17	F, 83	144	Loose stem, loose cup
18	F, 67	94	Loose stem, loose cup
19	M, 65	108	Loose stem, loose cup, osteolysis
20	M, 76	144	Loose stem, loose cup, osteolysis
21	M, 74	114	Loose stem, loose cup
22	M, 85	181	Loose stem, pain, osteolysis in acetabulum without loosening, but cup liner exchanged
23	F, 67	136	Loose stem, loose cup
24	M, 84	155	Loose stem, loose cup, fracture femur, osteolysis femur
25	M, 90	147	Loose stem, loose cup, osteolysis
26	F, 74	214	Loose stem, loose cup, fracture femur
27	M, 75	158	Loose stem, loose cup
28	F, 73	188	Loose stem, loose cup
Median	73.5^a^	137^b^	
Range	38–90	39–214	

^a^ The Mann–Whitney test was used to compare the medians of the cup and stem group (*p =* .36).

^b^ The Mann–Whitney test was used to compare the medians of the cup and stem group (*p* = .12).

### 2.2 Tissue preparation and histological evaluation

Periprosthetic tissue samples from the joint capsule or periprosthetic membranes were collected during revision surgery and fixed in 4% buffered formalin. The specimens were embedded in paraffin, sectioned at 4 μm and stained with hematoxylin and eosin (H&E). Two tissue slides per patient were selected for histological evaluation. However, in three cases, there was only one sample per patient available. Two pathologists (A. C. J. and K. S.) semi‐quantitatively evaluated the tissue slides from all patients in a blinded fashion using a modified Mirra classification^14^, ^15^ described by Doorn et al.^16^ Three cell‐rich high‐power fields (HPF, 40×) per tissue slide were counted by the two pathologists and the number of observations was divided by six and graded for macrophages, acute and chronic inflammatory cells, foreign body giant cells and the amount of metal particles. Hereby, the total number of cells and particle intensity was graded as absent (0), low (1+), moderate (2+), or high (3+), as described in our previous study.^15^ Additionally, in the present study, the degree of necrosis in the tissue sections was graded 0–10% (0), 10–50% (1+), 50–100% (2+), or 100% (3+). Tissue samples from three patients (case no. 6, 12, and 26) showed only connective tissue and/or fat tissue, which was not representative for the tissues to be analyzed. The histological results of these samples have been excluded from statistical analysis.

### 2.3 Particle characterization

Total wear particle load was assessed from the tissue slides with High‐Resolution Optical Darkfield Microscopy (HR‐ODM; Auburn, AL) as described before.^15^, ^17^ The particles in three particle‐rich sections of each tissue slide were counted and measured using image analysis software (NIS‐Elements 2.30, Nikon, Japan). Birefringent polyethylene (PE) particles were detected by light microscopy with a polarization filter and counted in three particle‐rich areas in 400× magnification per tissue slide (Olympus CX31, U‐TP530 Analyzer). Additionally, PE particles were isolated according to ISO 17853 in eight tissue samples. Following tissue digestion, the PE particles were purified by high‐speed centrifugation as described in ISO 17853, which allows all particle sizes (nm to several μm length) to be collected. The samples were then diluted up to 200 ml with deionized water and sequentially filtrated through membrane filters (Whatman, Ø 47 mm) with pore sizes of 10, 0.1 and 0.02 μm. The isolated particles were analyzed using a field emission scanning electron microscope (FE‐SEM Supra‐55VP, Carl Zeiss AG, Germany). SEM images were taken at 500× (10 μm filters) and 20,000× (0.1 and 0.02 μm filters). The median equivalent diameter, aspect ratio and size distribution of the PE particles were determined by measuring 100 particles per filter (only 0.1 and 0.02 μm) with image analysis software (NIS‐Elements 2.30, Nikon, Japan). Elongated particles, that is, threads, flakes or elliptical shapes, were considered as PE particles. Furthermore, 10 tissue sections were studied with field emission scanning electron microscopy (FE‐SEM, Merlin VP compact, Carl Zeiss AG, Germany) in secondary emission and back scattered modus at 15 kV. Briefly, the paraffin‐embedding was removed by soaking the samples in xylene (Merck, Darmstadt, Germany), followed by 100%, 96%, 80%, 70% ethanol (Arcus kjemi, Vestby, Norway) and finally rinsed in distilled water. The chemical composition of the particles in the tissue samples was determined using energy dispersive X‐ray (EDX) analysis.

### 2.4 Stem analysis

The damage of each retrieved stem was assessed using a semi‐quantitative grading system consisting of three abrasion levels (A, AA, AAA), described by Willert et al.^18^ No visible damage was defined as level 0. Each side of the stem was divided into four quadrants using the line between the rough and matt part as a natural division line between the proximal and distal part. The grades of each zone were added to give a total abrasion score of maximum 3 × 16 = 48 points.

### 2.5 Cup analysis

Using radiographs, the linear wear of the cup^19^ was determined with an X‐ray software “Hip X‐ray” using pre‐defined methods. Additionally, the orthopedic surgeons registered the Paprosky classification of femoral and acetabular bone loss^20^ from radiographs and intraoperative observations during revision surgery.

### 2.6 Blood analysis

Whole blood samples from 22 patients were analyzed as described previously.^15^ Briefly, blood samples were drawn just prior to revision surgery from the patient's forearm. An aliquot of 1.5 g whole blood was mixed with 3 ml 60% HNO_3_ and 2 ml 30% H_2_O_2_ and digested in a microwave‐assisted system (Milestone 1200 Mega, Sorisole Italy). A blank and Seronorm reference blood sample (Sero AS, Oslo, Norway) were treated in the same manner as the test samples. The concentration of Co, Cr, and Zi in the blood samples was determined by High‐Resolution Inductively Coupled Plasma‐Mass Spectrometry (HR‐ICP‐MS; Element 2 Thermo Scientific, Bremen, Germany).

### 2.7 Statistical analysis

Statistical analysis was done using GraphPad Prism 9 (GraphPad Software, La Jolla, CA). Data are presented as medians with range and CI, where applicable. Statistical differences between parameters (particle load/mm^2^, metal ion levels) in the independent groups were determined using the Mann–Whitney U test, which compares ranks of two different groups. Relationships between two non‐parametric parameters were analyzed using Spearman's rho correlation. Results were considered statistically significant with *p* < .05.

### 2.8 Ethics

The project protocol and the retrieval biobank were approved by the Regional Committee for Medical and Health Research Ethics (REC West, project number 2010/2817). The samples and patient information were coded and written informed consent from every patient prior to blood and tissue sampling was obtained.

## 3 RESULTS

The cases included 16 men and 12 women with a median age of 73 years (range, 38–90 years) at the time of revision surgery. Indications for primary THA included osteoarthritis (19 patients), avascular necrosis of the femoral head (3 patients), psoriatic arthritis (1 patient) and developmental dysplasia of the hip (1 patient). For 4 patients, there was no information available about the indication for primary THA. The main reasons for revision were cup loosening in 10 patients (cup group) and loosening of both stem and cup in 18 patients (stem group). Table 1 lists the patient data, implant duration, and reason for revision for the two groups. Additionally, a descriptive summary of the data (i.e., age of the patient at revision and implant duration) is given in Table 1.

### 3.1 Histology

**FIGURE 1:**
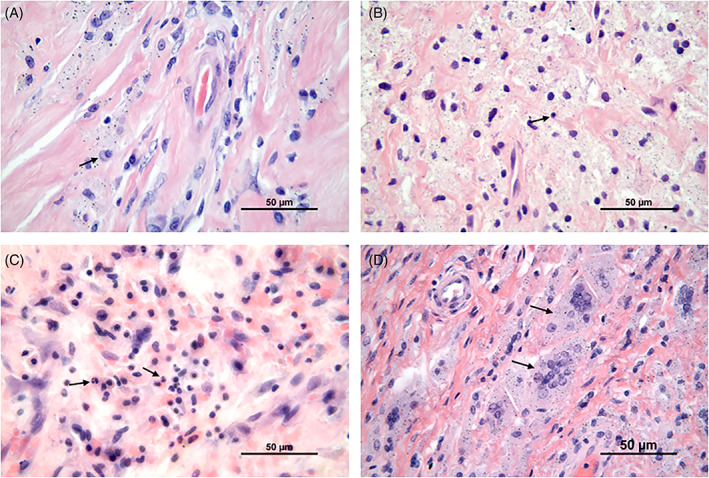
Light microscopy image of typical cells found in the periprosthetic tissue of both groups. (A) Macrophages with some plasma cells (black arrow), (B) Macrophages with some lymphocytes (black arrow), (C) Macrophages with scattered neutrophils, and (D) Multinucleated giant cells with phagocytosed wear particles. H&E ; Scale bar, 50 μm

**FIGURE 2:**
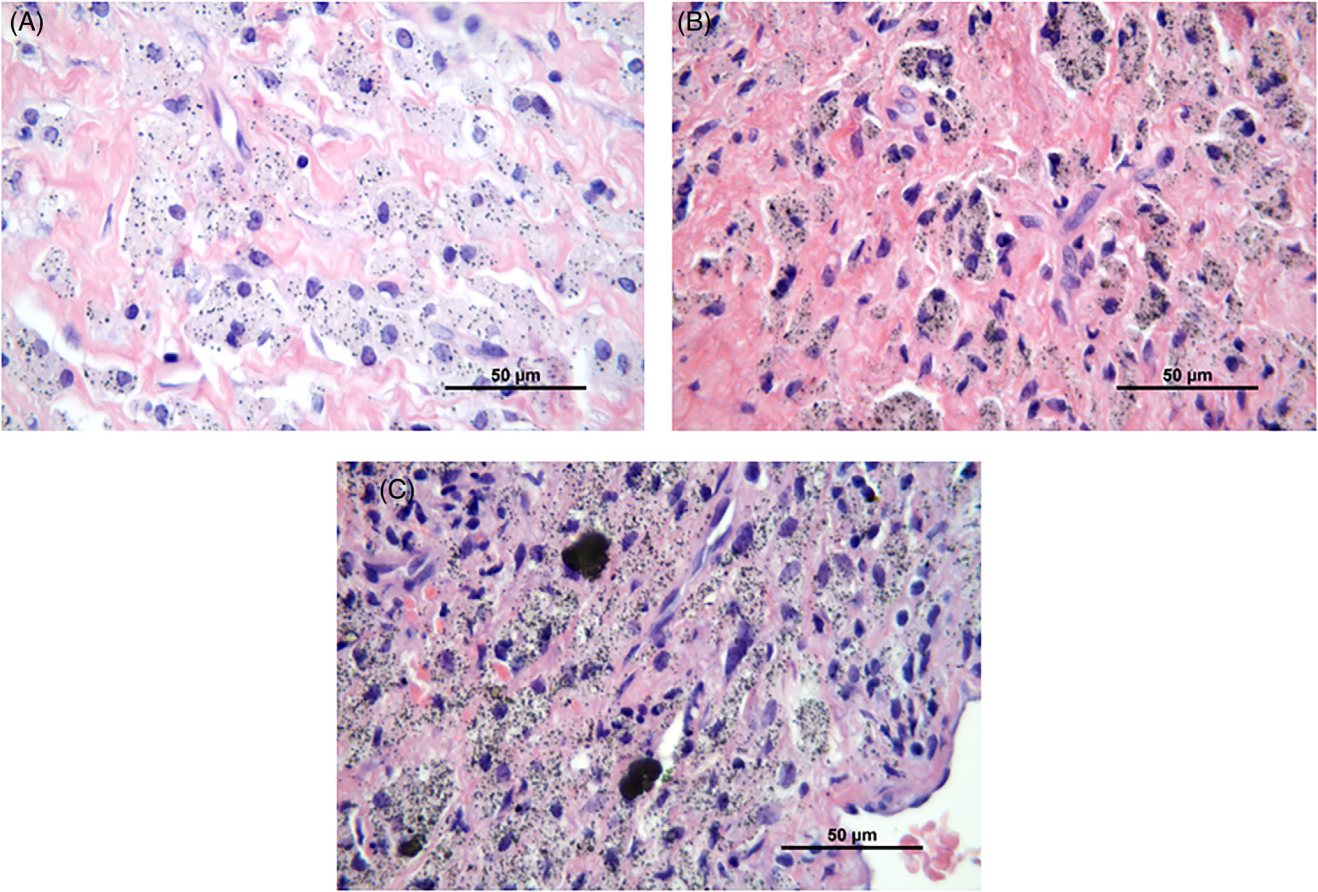
Wear particles phagocytosed by macrophages are seen as black and/or dark grey spots in the cytoplasm of these cells. The amount of wear particles in the macrophages was graded as (A) low (1+), (B) moderate (2+), and (C) high (3+). H&E, 63×

**FIGURE 3:**
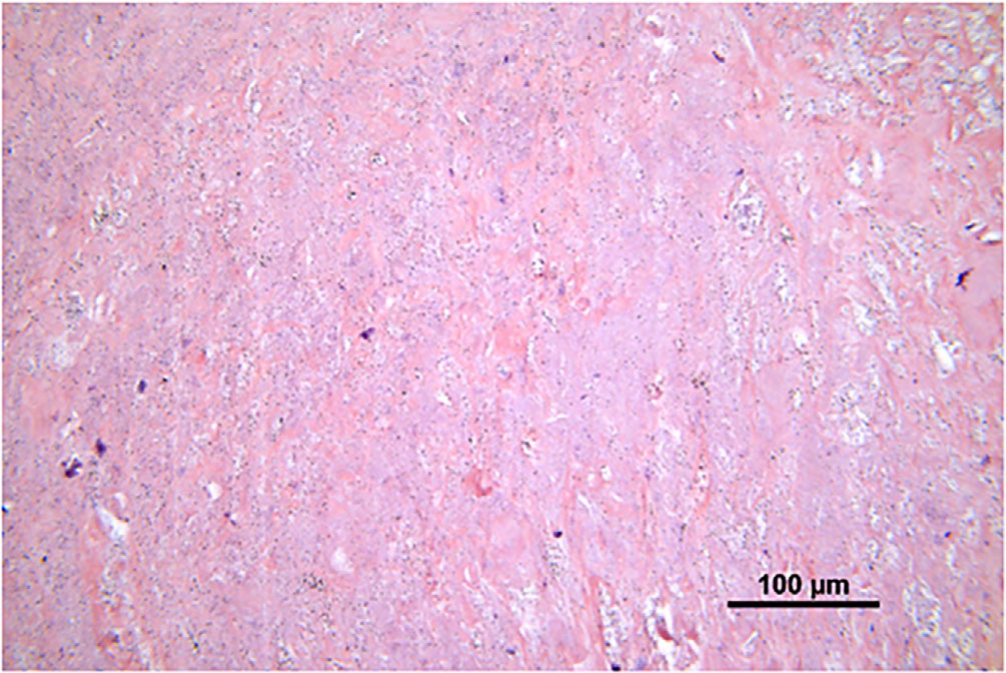
Necrosis of periprosthetic tissue. Paler stained, cell‐free tissue area, as compared to more cell‐rich areas as shown in Figure 1. H&E, 20×

Histological examination of the tissue samples revealed various amounts of macrophages, lymphocytes, neutrophils and multinucleated giant cells (Figure 1). The number of different cell types in the tissue samples from patients in the cup group and patients in the stem group was quite similar. Macrophages were the most prominent cell type in the periprosthetic tissues of both groups, and they were diffusely spread in the tissue (Figure 1A,B). A grade of 3+ was given in 58% and 50% of the cases in the stem group and cup group, respectively (Tables 2 and 3). A few plasma cells, but no lymphoid follicles were observed (Figure 1A). Chronic inflammatory cells, that is, lymphocytes, were mainly randomly distributed between the macrophages (Figure 1B), but not as numerous as these cells. A few scattered neutrophils were found in both groups (Figure 1C). Multinucleated giant cells, with grade 2+ or 3+, were found in 36% and 19% of the tissue samples in the stem group and the cup group, respectively (Figure 1D) (Tables 2 and 3). Wear particles were phagocytosed by macrophages and were seen as black and/or dark gray spots in the cytoplasm of these cells (Figure 2). Macrophages with metal particles were more abundant in tissue samples from patients in the stem group (median score 2+) than in patients in the cup group (median score 1+). Moreover, the number of macrophages with metal particles correlated with the total particle load/mm^2^ in this group (stem group: r_Sp_ = 0.407, *p =* .023, CI: 0.051–0.672; cup group: r_Sp_ = 0.221, *p =* .41, CI: −0.323 to 0.655). Necrosis, which appeared as paler stained, cell‐free tissue areas (Figure 3)—as compared to more cell‐rich areas (Figure 1)—was found in most of the tissue samples. The degree of necrosis correlated with the implant duration (in months; r_Sp_ = 0.337, *p =* .025, CI: 0.036–0.582). In the stem group, 48% of the cases were graded 2+ or 3+, which corresponds to 50 to 100% necrosis, whereas in the cup group, 43% of the tissue samples showed more than 50% necrotic areas.

**TABLE 2:** Periprosthetic tissue reactions in patients with cup loosening only (*n =* 10); evaluated with the modified Mirra classification

Case no.	Neutrophils	Macrophages	Chronic inflammatory cells	Multinucleated giant cells	Metal particles/macrophages	Necrosis
1‐1	0	2+	1+	0	2+	1+
1‐2	1+	3+	2+	0	2+	2+
2‐1	1+	3+	2+	0	1+	1+
3‐1	1+	3+	3+	3+	1+	1+
3‐2	2+	2+	2+	1+	1+	1+
4‐1	1+	3+	2+	0	1+	0
4‐2	1+	3+	1+	0	2+	0
5‐1	2+	3+	2+	3+	0	2+
5‐2	0	0	0	0	0	3+
6‐1	NR^a^					
6‐2	NR^a^					
7‐1	0	2+	2+	0	1+	2+
7‐2	0	3+	1+	1+	0	0
8‐1	0	0	0	0	0	3+
8‐1	1+	2+	1+	1+	2+	1+
9‐1	0	3+	1+	3+	1+	0
10‐1	0	0	0	0	0	3+
10‐2	0	0	0	0	0	3+

Frequency distribution (%) and medians of scores 0 to 3+						
	Neutrophils	Macrophages	Chronic inflammatory cells	Multinucleated giant cells	Metal particles/macrophages	Necrosis
Score	Frequency distribution (%)					
0	50	25	25	62.5	37.5	25
1+	37.5	0	31.25	18.5	37.5	31.25
2+	12.5	25	37.5	0	25	18.75
3+	0	50	6.25	18.75	0	25
Median score	0.5+	2.5+	1+	0	1+	1+

^a^ NR, not representative. Tissue that is not representative, such as connective tissue or fat tissue. These samples were excluded from the histological examination.

**TABLE 3:** Periprosthetic tissue reactions in patients with stem and cup loosening (*n =* 18); evaluated with the modified Mirra classification

Case no.	Neutrophils	Macrophages	Chronic inflammatory cells	Multinucleated giant cells	Metal particles/macrophages	Necrosis
11‐1	2+	2+	2+	2+	2+	2+
11‐2	1+	3+	2+	1+	2+	1+
12‐1	NR^a^					
12‐2	1+	3+	2+	0	1+	1+
13‐1	1+	3+	1+	0	2+	1+
13‐2	0	0	0	0	0	3+
14‐1	0	2+	1+	0	2+	1+
14‐2	0	3+	2+	0	2+	2+
15‐1	0	3+	2+	2+	2+	0
15‐2	0	3+	2+	2+	2+	0
16‐1	0	2+	1+	0	2+	2+
16‐2	0	3+	2+	0	2+	2+
17‐1	1+	2+	2+	0	2+	0
17‐2	1+	3+	2+	0	2+	0
18‐1	1+	3+	2+	0	1+	1+
18‐2	1+	3+	2+	2+	2+	1+
19‐1	0	3+	1+	2+	2+	0
19‐2	0	2+	2+	2+	2+	0
20‐1	0	3+	2+	1+	1+	1+
20‐2	0	3+	2+	2+	2+	1+
21‐1	1+	3+	1+	3+	2+	1+
22‐1	0	0	0	0	0	3+
23‐1	0	3+	2+	3+	1+	2+
23‐2	1+	3+	2+	2+	1+	2+
24‐1	0	0	0	0	0	3+
24‐2	0	0	0	0	0	3+
25‐1	0	0	0	0	0	3+
25‐2	0	0	0	0	0	3+
26‐1	NR^a^					
26‐2	NR^a^					
27‐1	1+	3+	2+	3+	2+	1+
27‐2	0	3+	2+	0	2+	2+
28‐1	1+	2+	1+	0	1+	2+
28‐2	1+	2+	1+	0	2+	2+

Frequency distribution (%) and medians of scores 0 to 3+						
	Neutrophils	Macrophages	Chronic inflammatory cells	Multinucleated giant cells	Metal particles/macrophages	Necrosis
Score	Frequency distribution (%)					
0	58.1	19.4	19.4	58.1	19.4	19.4
1+	38.7	0	22.6	6.5	19.4	32.3
2+	3.2	22.6	58.1	25.8	62.3	29.0
3+	0	58.1	0	9.7	0	19.4
Median score	0	3+	2+	0	2+	1+

^a^ NR, not representative. Tissue that is not representative, such as connective tissue or fat tissue. These samples were excluded from the histological examination.

### 3.2 Particle analysis

**FIGURE 4:**
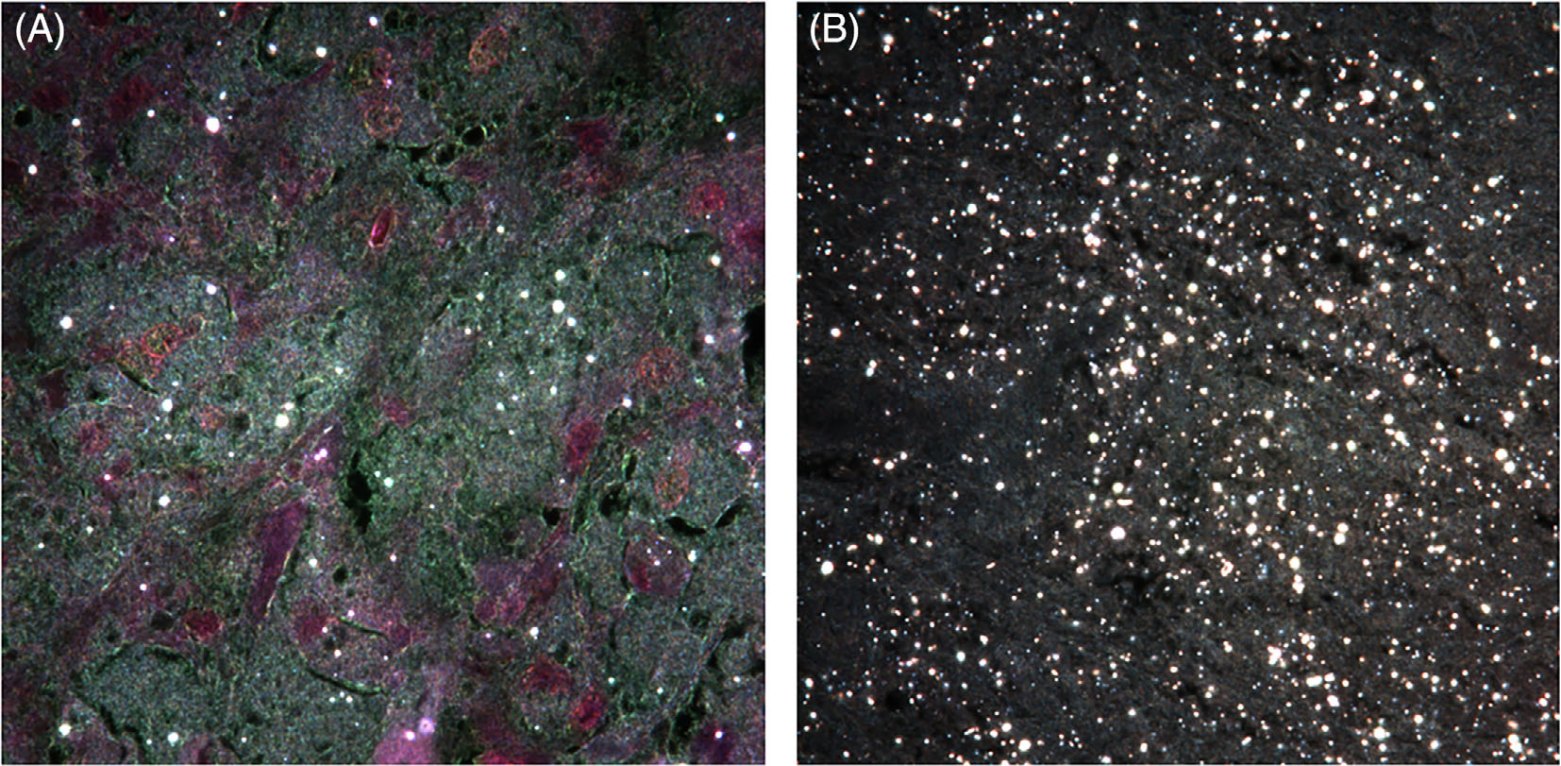
High resolution darkfield microscopy images (H&E, 100×) showing tissue samples from the cup group (A) and stem group (B) containing wear particles (including CoCrMo, ZrO_2_, and PE) which appear white. Representative image from each study group

**FIGURE 5:**
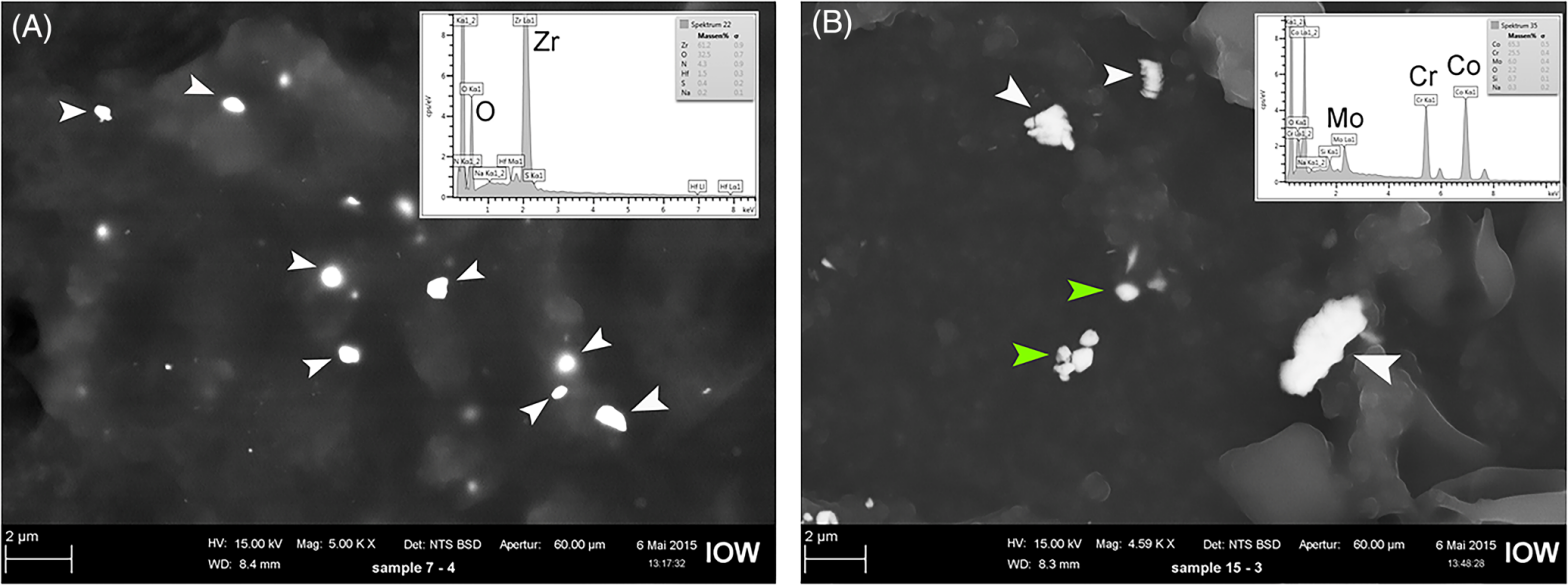
Scanning electron microscopy (SEM) images with EDXA spectra of isolated particles. (A) SEM image; backscattered mode; highlighting ZrO_2_ particles (white arrows) found in capsule tissue from case no. 2. (B) SEM image; backscattered mode; highlighting CoCrMo particles (white arrows) found in tissue in the femur region from case no. 12. Green arrows show BaSO_4_ particles found in the cement used in this case

**FIGURE 6:**
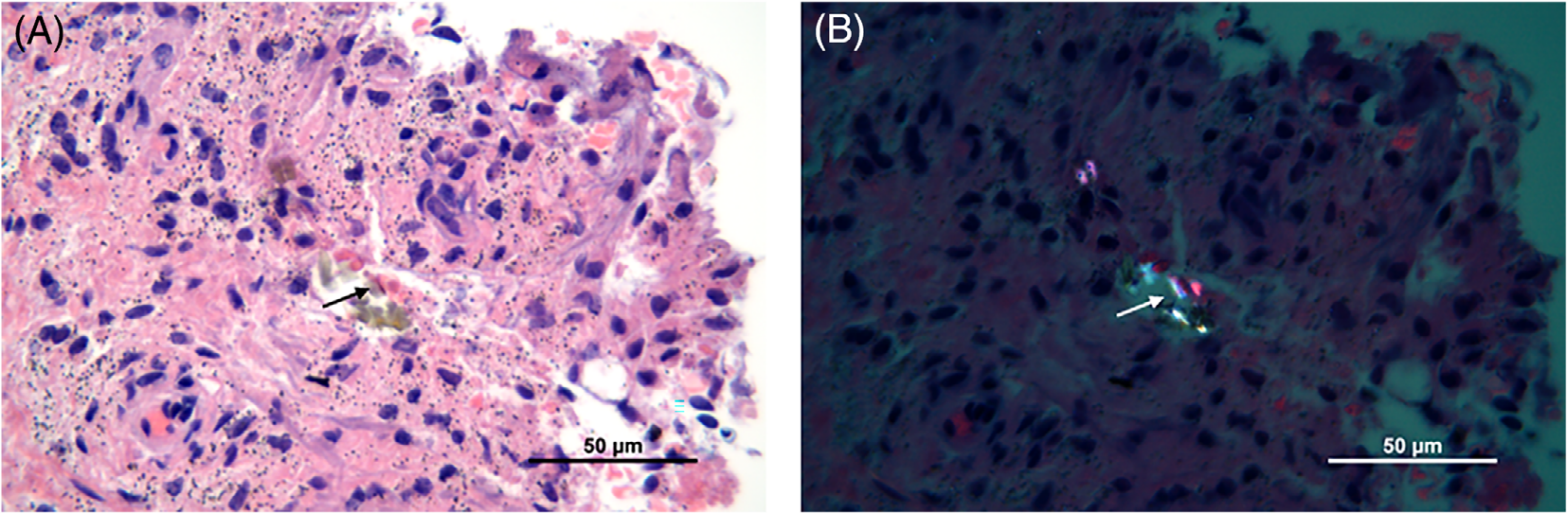
Light microscopy image of different wear particles in periprosthetic tissue. (A) A mixture of metal particles and PE particles (black arrow). (B) Birefringent PE particles (white arrow) visualized with a polarizing filter. H&E, 63×

Using high‐resolution optical microscopy, a higher total particle load was found in tissue samples from patients in the stem group compared to the samples from the cup group (median stem = 23,893 particles/mm^2^, range: 8127–83,021 particles/mm^2^, vs. median cup = 9244 particles/mm^2^, range: 232–55,362 particles/mm^2^; *p =* .001) (Figure 4). SEM‐EDXA showed that zirconium dioxide (ZrO_2_) particles from the bone cement were frequent in the tissue samples and these particles were usually round and below 2 μm (Figure 5A). CoCrMo particles were mainly found in the stem‐related tissue sections and were normally longer than 1 μm and irregular in shape (Figure 5B). Using polarization microscopy, PE particles were found in most of the tissue samples from both groups (Figure 6B) (median stem group = 1.65 PE/mm^2^, range: 0–52.26 PE/mm^2^; median cup group = 0.35 PE/mm^2^, range: 0–21.9 PE/mm^2^, *p =* .149). Analysis of isolated PE particles by SEM showed a high number of submicron PE particles. The median equivalent diameter was 0.31 μm with a range from 0.05 μm to 1 μm. The shape often appeared as rolled threads/twisted flakes (Figure S1).

### 3.3 Implant analysis

**FIGURE 7:**
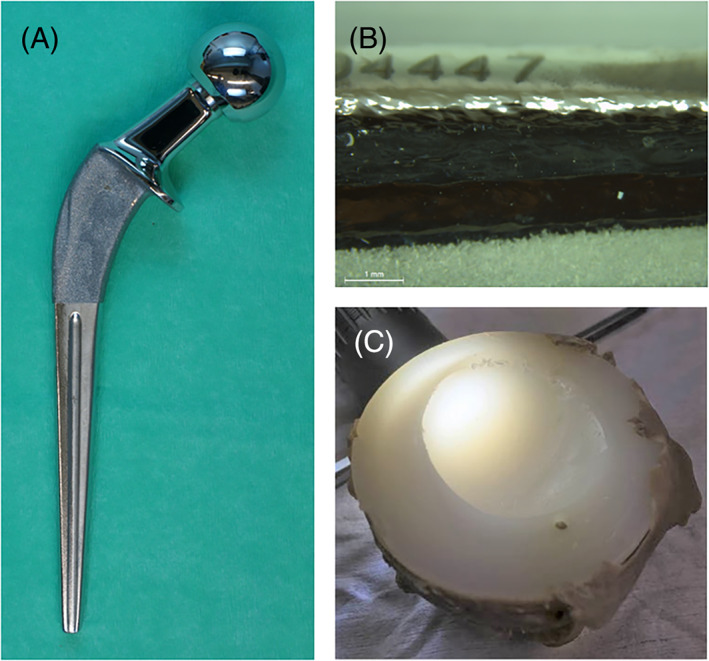
(A) Femoral stem abrasion and polishing (femoral stem from case 24). (B) Typical stem damage on the posterior‐distal‐medial side with grade AAA abrasion and polishing (femoral stem from case 24). (C) Cup wear (cup from case 5)

Stem analysis showed that most of the stems had signs of mechanical wear, seen as abrasion and polishing (Figure 7A,B). In 44% of the cases, the stems were highly worn (abrasion score ˃ 30). Femoral bone loss was found to be extensive (Paprosky type II and III) in 15 of 18 cases (Table 4). The median linear wear of 10 cups in the stem group was 2.07 mm (range: 0.90–4.05 mm) and the wear rate 0.24 mm/year (range: 0.11–0.36 mm/year). In the stem group, acetabular bone loss was found to be extensive in 83% of cases (15 out of 18 cases; Table 4). Cup analysis showed that most cups were highly worn (Figure 7C). Third‐body wear particles, mostly made of ZrO_2_ and some CoCrMo, were found inside the cups, embedded in the plastic surface. In the cup group, the median linear wear of the cups was 1.60 mm (range: 0.82–3.31 mm) and the wear rate 0.21 mm/year (range: 0.11–0.32 mm). Acetabular bone loss was extensive (Paprosky type II and III) in 80% of the cases of the cup group (8 out of 10 cases; Table 4).

**TABLE 4:** Implant and wear analysis and classification of femoral and acetabular bone loss (Paprosky score) in patients with loose cup only and in patients with loose stem with cup loosening

Case no.	Abrasion score stem	Total linear wear cup (mm)	Linear wear/year (mm)	Paprosky score acetabulum	Paprosky score femur
*Loose cup only*					
1		0.92	0.14	2	0
2		1.71	0.27	3	0
3		1.20	0.16	1	1
4		1.85	0.32	2	0
5		2.41	0.22	2	0
6		1.26	0.20	2	0
7		1.49	0.24	1	1
8		0.82	0.11	2	0
9		2.79	0.32	2	0
10		3.31	0.20	2	0
*Loose stem and cup*					
11	32	1.94^a^	0.24	3	2
12	28	2.20	0.20	3	3
13	31	1.23	0.16	2	2
14	32	0.90	0.11	2	1
15	35	1.17^a^	0.36	2	2
16	30	1.30	0.18	1	2
17	19			1	1
18	28	2.42	0.31	2	2
19	30			2	3
20	23	4.05	0.35	2	2
21	25	2.72	0.29	2	2
22	25			0	3
23	9			2	2
24	21			2	3
25	18			3	0
26	13			3	3
27	34			2	3
28	35	2.59	0.24	2	2

^a^ No postoperative X‐ray available. Values have been calculated by multiplying linear wear/year by years implanted.

### 3.4 Metal ion analysis

**FIGURE 8:**
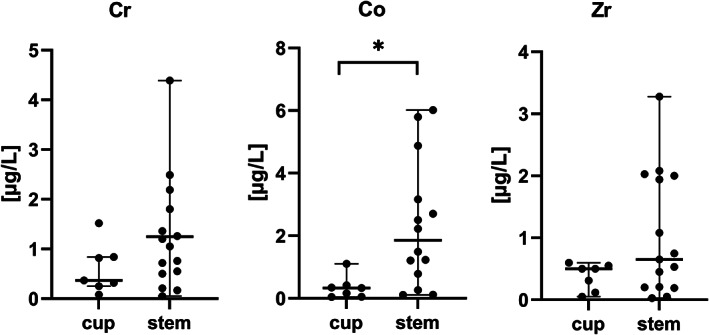
Metal concentrations in blood samples, μg/L (medians and range). (*) significant difference between cup and stem group, *p* ˂ .05

The median concentrations of chromium, cobalt and zirconium in whole blood samples (*n =* 22) from all patients included in this study were 0.79, 0.94, and 0.51 μg/L, respectively. The median concentrations of chromium in blood samples in patients in the stem group (*n =* 15) were 1.05 μg/L (range: 0.05–4.39 μg/L) and higher than for those in the cup group (*n =* 7; 0.37 μg/L, range: 0.08–1.52 μg/L), although not significant (*p =* .23) (Figure 8). The median concentrations of cobalt in blood samples from patients in the stem group (1.85 μg/L, range: 0.11–6.02 μg/L) were significantly higher than in blood samples from patients in the cup group (0.33 μg/L, range: 0.04–1.1 μg/L), with *p =* .0097. The median concentration of zirconium in blood samples from the stem group was 0.65 μg/L (range: 0.03–3.28 μg/L), while it was 0.50 μg/L (range: 0.05–0.60 μg/L) in the cup group, (*p =* .21) (Figure 8). The concentration of Zr in blood correlated (r_Sp_ = 0.639, *p =* .026) with the Paprosky score. The concentrations of Cr (r_Sp_ = 0.758, *p =* .002), Co (r_Sp_ = 0.588, *p =* .023) and Zr (r_Sp_ = 0.720, *p =* .003) correlated with the total stem abrasion in the samples from patients with loosening of stem and cup. In this group, the concentrations of Cr (r_Sp_ = 0.417, *p =* .027) and Co (r_Sp_ = 0.535, *p =* .005) in blood correlated with the total particle load/mm^2^. All blood ion levels significantly correlated to the total particle load/mm^2^ in all tissue samples taken together (r_Sp_[Co] = 0.386, *p =* .012; r_Sp_[Cr] = 0.540, *p =* .0003; r_Sp_[Zr] = 0.343, *p =* .028).

## 4 DISCUSSION

In the present study, we aimed to assess inflammatory tissue responses, particle characteristics in the peri‐prosthetic tissues, implant wear and blood metal ion levels in patients revised for loose components of a specific cemented THA (Spectron EF stem and Reflection non‐crosslinked All‐Poly cup). Implant degradation products due to wear and corrosion are considered a critical factor in the onset of osteolysis and subsequent aseptic loosening of hip implants.^21^, ^22^ Depending on the particle characteristics (size, shape and composition),^15^, ^23^ wear particles (metal, PE and bone cement particles) and metal ions in the periprosthetic tissue provoke an inflammatory environment, which is characterized by macrophages and T cells in the tissue, that are occasionally accompanied by multinucleated giant cells and eosinophils.^24^ Our results showed that the tissue samples in both groups contained predominantly macrophages with lymphocytes mainly randomly distributed between the macrophages. Generally, adverse local tissue reactions (ALTR) with a large number of macrophages present little lymphocyte infiltration, and lesions that are highly lymphocyte‐infiltrated do not exhibit a large number of macrophages.^25^ There were more macrophages than lymphocytes in the periprosthetic tissue samples from both the cup and the stem group. This is in contrast to a recent study that showed that the macrophage pre‐dominant pattern in the periprosthetic tissue is usually associated with Metal‐on‐Metal (MoM) hip prostheses, whereas a mixed macrophage‐lymphocyte pattern was found to be more frequent in tissue samples from Metal‐on‐Polyethylene (MoP) hip implants.^26^ At the femoral side, wear particles of CoCrMo, polymethylmethacrylate (PMMA) and the radiopacifier ZrO_2_ were found. Activated macrophages phagocytize these wear particles, which is evidenced by the particles within these cells (Figure 3). Macrophages containing metal and cement particles were more frequently found in tissue samples from patients with stem loosening. Moreover, the number of macrophages containing wear particles correlated to the total particle load, an indirect marker of wear in this group. This finding supports the results of a previous study by Campbell et al. that demonstrated a correlation between the number of macrophages in the tissue and wear of the femoral stem.^27^ The low number of neutrophils in the tissue samples suggests that direct neutrophil mediated innate immune responses only play a minor role in the pathogenesis of ALTR in the patients in our study. Most of the tissue samples in our study showed large areas of necrotic tissue, which is in agreement with a study by Eltit et al.^28^ Moreover, tissue necrosis was found to be proportional to the implant duration. This finding is not surprising, as implant wear progresses over time, the periprosthetic tissue gets more destructed by the inflammatory mediators and eventually cell death occurs. We also identified PE particles in the periprosthetic tissues in both groups. At the acetabular side, UHMWPE particles are released through the head penetration into the cup during movement. Surprisingly, we detected a slightly higher number of PE particles in the tissue samples from the stem group compared to the cup group. However, due to detection limitations with polarized light microscopy, we also did SEM analysis of isolated PE particles that showed a high number of submicron PE particles in both groups that were too small to be seen with light microscopy. The median diameter of the PE particles was found to be 0.31 μm (range 0.05–1 μm), which is slightly less than the mean diameter (660 nm) reported by a previous study.^21^ However, the small sample size in both groups made statistical analysis unfeasible. Thus, it remains unclear if the tissue samples in the stem group contained more PE particles than samples in the cup group. Implant‐derived metal ions have also been shown to play a pivotal role in the osteolytic process.^29^ Metal ions can be released by multiple mechanisms: (a) wear‐mediated disruption of the passive layer on the implant surface, (b) release of metal ions from nano‐ and micron‐sized particles in body fluids, (c) cellular reactions—especially in the lysosomes—to the phagocytized particles generate metal ions that can be released into the periprosthetic tissue.^25^ Several studies have shown the inflammatory and osteolytic effect of cobalt and chromium ions.^30^, ^31^ The presence of elevated metal ions in synovial fluid and serum has been associated with the development of adverse local reactions in periprosthetic tissue.^32^ A previous study demonstrated a strong correlation between elevated metal ion concentrations and patients with ALTR.^33^ Paukkeri et al.^34^ showed that patients with a macrophage‐dominated response had significantly higher blood chromium and cobalt ion levels compared to patients with T‐lymphocyte‐dominated response. Conversely, but in agreement with other previous studies,^35^, ^36^ we did not find any correlation between the histological findings, that reflect inflammatory responses and necrosis in the periprosthetic tissue, and blood metal ion content. However, all these studies assessed MoM‐prostheses, while MoP hip implants were analyzed in the current study. Cobalt and chromium serum ion levels in patients with MoP hip implants are far less studied than in patients with MoM hip implants. Metal ion levels in well‐functioning MoP hip implants are commonly used as a control for patients with MoM hip implants.^37^ Nevertheless, a threshold of 1 μg/L as cutoff for the identification of ALTR in patients with MoP hip implants was proposed recently,^38^ as compared to the threshold of 7 μg/L in MoM hip implants. Thus, the threshold used in MoM bearing patients cannot be translated to MoP bearing patients. Our analysis showed that patients with stem loosening had a median cobalt concentration of 1.85 μg/L in blood. The median chromium ion levels in this group were equal to the proposed threshold of 1 μg/L (1.05 μg/L). Although there was no clear correlation between the blood metal ion levels and the histological findings, our results support the above‐mentioned findings by Fillingham et al.^38^ In both groups, the blood cobalt ion concentration was higher than that of chromium. Chromium is known to accumulate to a high degree in the periprosthetic tissue while cobalt ions are rapidly transported to the blood stream and are eliminated via urine.^36^ We found that blood metal ion concentrations correlated with the total particle load and stem abrasion, which is not surprising given the roughness of the proximal stem. Similarly, a recent analysis showed elevated Cr ion levels in serum with increasing roughness of CoCrMo femoral head in MoP hip implants.^28^

As a result of micromotion at the interface between the stem's rough surface and the cement, metallic and cement particles are generated due to fretting and abrasion.^39^, ^40^ These particles, together with the PE particles, originating from the cup, are capable of causing osteolysis and loosening of the stem.^5^, ^6^ A potential consequence of the activation by, and phagocytosis of, wear particles is cellular toxicity, including cell death. Previous studies have shown that PE and metal particles can induce apoptosis in macrophages in a size‐ and dose‐dependent manner.^41^ However, it has also been demonstrated in cell culture studies, that macrophages stay viable and are able to tolerate PE particles better than metal particles, which led to an increased osteoclast differentiation (favoring osteolysis) of PE‐laden macrophages compared to macrophages that phagocytized CoCrMo particles.^21^, ^42^ High PE wear, due to the inferior plastic quality, led to osteolysis in the acetabulum and cup loosening. The wear rate is comparable to findings from radiostereometric (RSA) studies with the same cup type of non‐crosslinked UHMWPE.^10^ Taken together, the design of the Spectron EF stem with its high proximal roughness gives an excessive amount of metallic and cement wear particles during stem loosening. Furthermore, the hard cement particles also increase cup plastic wear through “third‐body wear”. The resulting inflammatory tissue responses due to the high particle load leads to excessive osteolysis around the implants in patients with the Spectron EF stem and the Reflection All‐Poly cup.

## Supporting information


**Figure S1**: Scanning electron microscopy (SEM) image of isolated PE particles (red arrows) on a membrane filter (0.1 μm pore size).

## Data Availability

The data that support the findings of this study are available from the corresponding author upon request.
