# Supplementary figures and images for: Inflammatory tissue reactions around aseptically loose cemented hip prostheses: A retrieval study of the Spectron EF stem with Reflection All‐Poly acetabular cup
Source: J Biomed Mater Res B Appl Biomater. 2022 Jan 31;110(7):1624–36. doi: 10.1002/jbm.b.35023 (PMC9303329; doi:10.1002/jbm.b.35023)

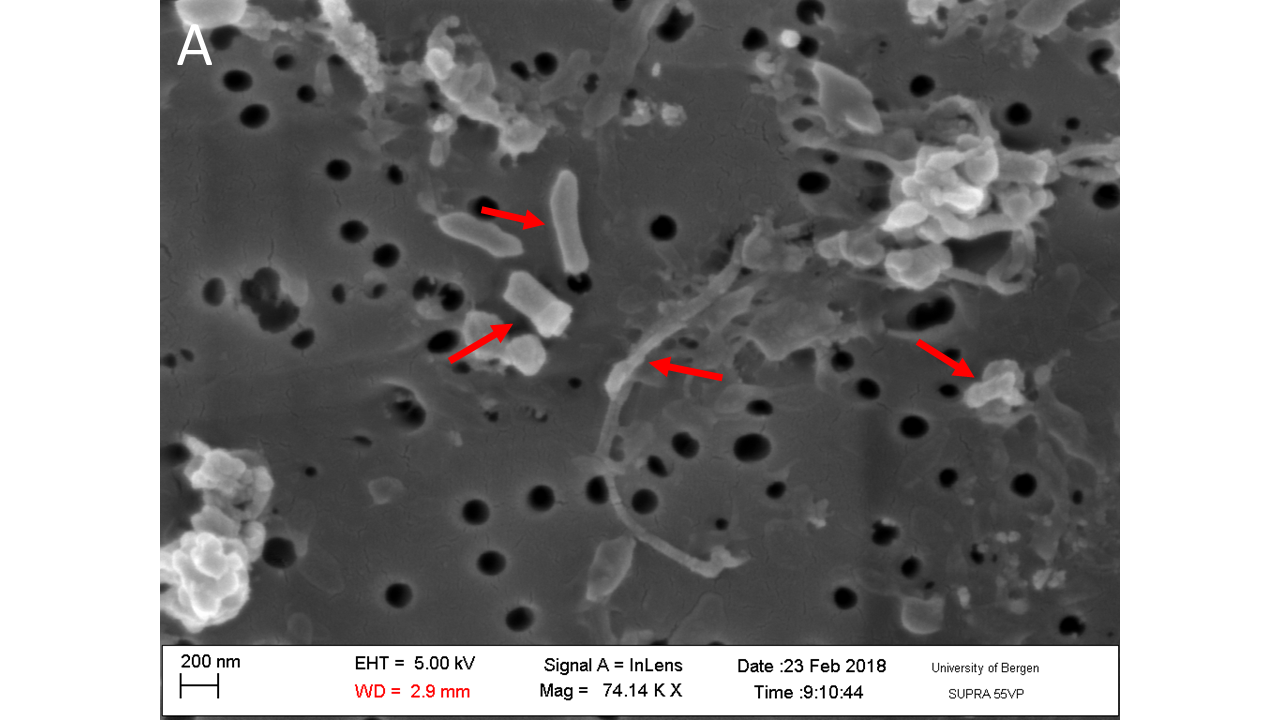

Supplement: Supplementary file 1 — Figure S1: Scanning electron microscopy (SEM) image of isolated PE particles (red arrows) on a membrane filter (0.1 μm pore size). [file JBM-110-1624-s001.tif]
